# Protein-Based Flexible Conductive Aerogels for Piezoresistive Pressure Sensors

**DOI:** 10.1021/acsabm.2c00348

**Published:** 2022-06-13

**Authors:** Yusheng Yuan, Niclas Solin

**Affiliations:** † Department of Physics, Chemistry, and Biology, Biomolecular and Organic Electronics, Linköping University, 581 83 Linköping, Sweden

**Keywords:** conductive polymers, gelatin, protein fibrils, aerogels, piezoresistive pressure sensor

## Abstract

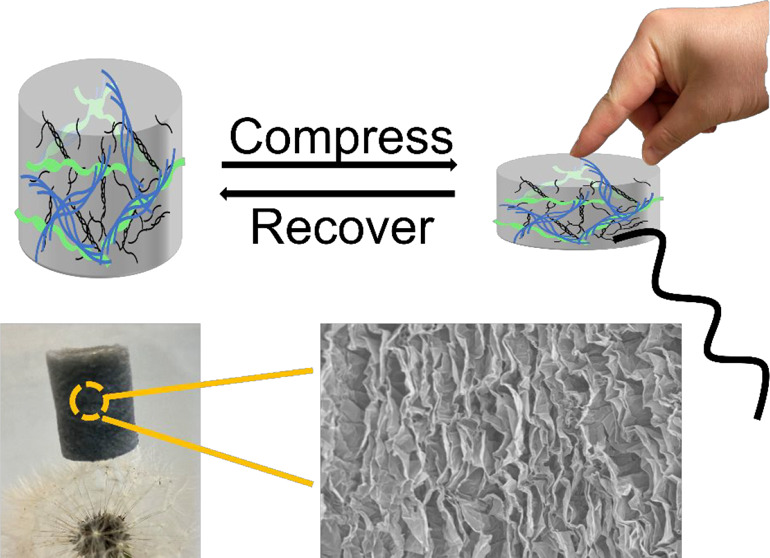

Gelatin is an excellent gelling agent and is widely employed for hydrogel formation. Because of the poor mechanical properties of gelatin when dry, gelatin-aerogels are comparatively rare. Herein we demonstrate that protein nanofibrils can be employed to improve the mechanical properties of gelatin aerogels, and the materials can moreover be functionalized with a an electrically conductive polyelectrolyte resulting in formation of an elastic electrically conductive aerogel that can be employed as a piezoresistive pressure sensor. The aerogel sensor shows a good linear relationship in a wide pressure range (1.8–300 kPa) with a sensitivity of 1.8 kPa^–1^. This work presents a convenient way to produce electrically conductive elastic aerogels from low-cost protein precursors.

## 1 Introduction

Gelatin has been widely used in the food, pharmaceutical, and cosmetic industries due to its biodegradability, biocompatibility, and nonimmunogenicity.^1−4^ Gelatin offers many special properties that are not easily imitated by other hydrocolloids, the most prominent being its excellent ability to form thermally reversible hydrogels. An interesting approach is to try to modify the properties of gelatin by forming hybrids with other materials.^5−8^ It has for example been shown that addition of polysaccharides like alginate, agar, carrageenan, or cellulose in gelatin-based gels affects rheological properties of the resulting hybrid materials.

A type of gel that has received much recent interest is aerogels^9,10^ including aerogels derived from biomass.^11,12^ Aerogels are solid materials often formed by replacing the water present in a hydrogel with a gas, typically air, while leaving the solid network of the gel intact. The development of novel methods for aerogel preparation have resulted in a range of novel low-density materials suitable for a wide range of applications including energy storage and conversion,^13−15^ sensing,^16,17^ catalysis,^18,19^ and environmental remediation.^20−22^ There is much recent interest in development of sensors that can detect mechanical deformations,^23−29^ and aerogels and foams have also been developed for such applications.^26−28^ In contrast to its popularity for forming hydrogels, employment of gelatin for preparation of aerogels is comparatively rare, and early studies focused on building 3D structures suitable for tissue engineering.^30^ In recent years, gelatin-based aerogels have been investigated as adsorbents (e.g., for oil or metal ions).^31,32^ Recently, some examples of gelatin-based aerogels with elastic properties have been reported, employing mixtures of gelatin and polymeric materials like poly(vinyl alcohol) (PVA) or cellulose or conductive materials (graphene oxide or Mxene).^33−36^

Fiber-based materials are investigated as components in hybrid materials for a wide range of applications.^37,38^ An interesting candidate for preparation hybrid materials with gelatin includes protein nanofibrils (PNFs). PNFs are formed by self-assembly of proteins, typically when heated in acidic solutions.^39−43^ PNFs have lengths in the μm range and diameters up to about 10 nm.^44^ A wide range of proteins have been demonstrated to form PNFs, including proteins isolated from plants and industrial side streams,^45^ and many interesting materials systems incorporating PNFs have been developed.^46−50^ An interesting approach for formation of electrically conductive PNF materials is functionalization with conductive polymers. This can be achieved either by polymerization of the respective monomer in the presence of PNFs,^51,52^ or by complexation with electrically conductive polyelectrolytes,^53−55^ in both cases giving electrically conductive hybrid materials. Herein we employ poly(4-(2,3-dihydrothieno[3,4-*b*]-[1,4]dioxin-2-yl-methoxy)-1-butanesulfonic acid (PEDOT-S)^56,57^ as a conductive polymer to functionalize hybrids between gelatin and lysozyme protein nanofibrils (LPNF). PEDOT-S has been investigated as a functionalization agent for PNFs,^53−55^ DNA,^58^ and liposomes^59^ to prepare electrically conductive hybrids materials.

**Figure 1:**
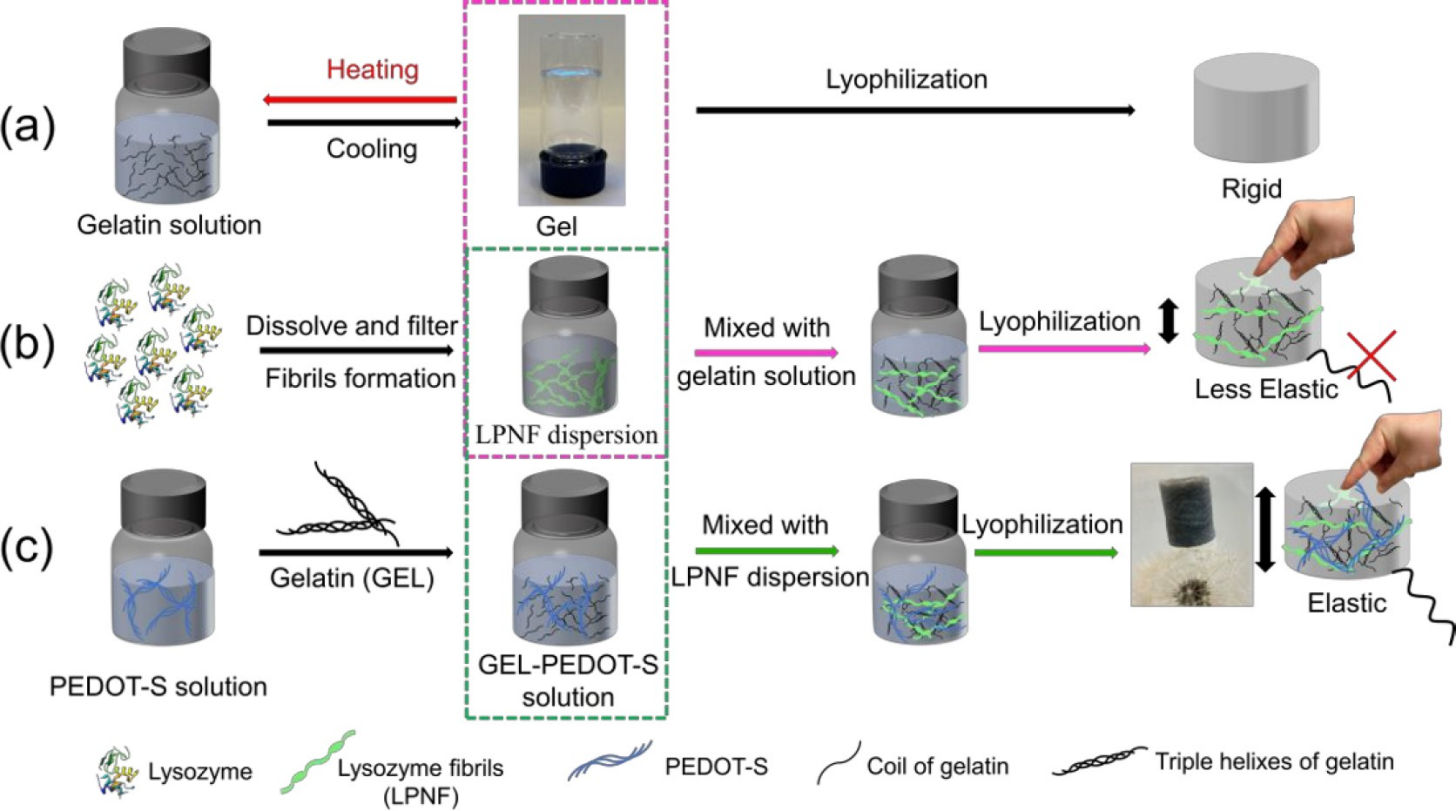
Schematic illustration of the procedures employed for preparation of aerogels from different combinations of gelatin, LPNFs, and PEDOT-S. (a) Preparation from pure gelatin. (b) Preparation from gelatin and LPNFs. (c) Preparation from gelatin, PEDOT-S, and LPNFs.

PNFs can be conveniently prepared by self-assembly of hen egg white lysozyme, a protein that is available at low cost in large quantities (employed as a food preservative and by brewers). We find that elastic electrically conductive aerogels can be formed from mixtures of lysozyme PNFs (LPNFs), gelatin, and PEDOT-S. A schematic illustration is provided in Figure 1. Briefly, a reference gelatin hydrogel was prepared and then lyophilized to form a rigid aerogel. Alternatively, LPNFs were mixed with either a gelatin solution, or with or gelatin:PEDOT-S solution, and the resulting mixture was cooled to induce gelation. Aerogels were then obtained by freezing of the hydrogel followed by evaporation under reduced pressure (freeze-drying). The various gels were investigated regarding their mechanical and electrical properties, and it was found that hybrids of LPNFs, gelatin, and PEDOT-S can form elastic electrically conductive aerogels that can function as pressure sensors.

## 2 Materials and Methods

### 2.1 Materials and Instruments

Hen egg-white lysozyme (HEWL) was purchased from Kemikalia AB, Sweden. Na_2_S_2_O_8_, K_2_S_2_O_8_, FeCl_3_, and HCl were purchased from Sigma-Aldrich. Gelatin was obtained from Sinopharm Chemical Reagent Co., Ltd. All chemicals were used as received without further purification, and doubly distilled water (18.2 Ω) was used throughout. Scanning electron microscopy (Zeiss Sigma 300 VP, Germany) with an acceleration voltage of 5 kV was used to obtain SEM images after painting Pt on the aerogels for 10 s. A biotrode pH meter (Hamilton Bonaduz AG, Switzerland) was used to measure the pH values.

### 2.2 PEDOT-S Synthesis and Purification

PEDOT-S was synthesized according to the procedure developed by R. Karlsson et al.^56^ Briefly, EDOT-S (0.2 g) was dissolved in water (3 mL). A mixture of K_2_S_2_O_8_ (0.29 g) (or Na_2_S_2_O_8_) and FeCl_3_ (0.005 g) dissolved in water (3 mL) was dropwise added to the stirred EDOT-S solution. After 4 h, the reaction was quenched by dilution with acetone (40 mL). When the product precipitated, it was centrifuged (5 min, 3500 rpm). The collected precipitate was dissolved in water (7 mL) and precipitated from acetone (40 mL). The procedure was repeated twice. Finally, the polymer was dialyzed against deionized water for 48 h using 800 g/mol cutoff membrane (Spectra/Por) and freeze-dried prior to use. Yield ∼45% (with respect to monomer unit).

### 2.3 Preparation and Self-assembly of LPNF Matrix

The hen egg-white lysozyme (HEWL, 2 wt %) was dissolved with 25 mM hydrochloric acid followed by filtration through a 0.45 μm poly(ether sulfone) (PES) filter. The resulting dispersion was heated at 80 °C with magnetic stirring at 1000 rpm for 24 h to obtain the fibrils dispersion.

### 2.4 Preparation of LPNF Co-assembled Hydrogels and Aerogels

#### 2.4.1 Preparation of LPNF:GEL Hydrogels and Aerogels

Gelatin (0.4 g) was dissolved in 20 mL of double-distilled water and stirred for 1 h at 60 °C until the gelatin was completely dissolved, which resulted in a 2 wt % gelatin solution. The 2 wt % LPNF dispersions were mixed with gelatin solutions in different volume ratios such as 3:1, 2:2, or 1:3 (meaning that the total amount of protein material was kept constant) by magnetic stirring for 10 min, and then the obtained mixture was maintained at 4 °C for 2 h, forming emulsions-gels. The resulting hydrogels were frozen at −80 °C overnight and lyophilized for 48 h to remove water, giving the aerogels.

#### 2.4.2 Preparation of LPNF:GEL:PEDOT-S Hydrogels and Aerogels

Gelatin (0.4 g) was dissolved in 20 mL of 0.25 wt % PEDOT-S aqueous solution, and the resulting mixture was stirred for 1 h at 60 °C until the gelatin was completely dissolved (giving a GEL:PEDOT-S: 8:1 solution). Then the 2% LPNF dispersions were mixed with GEL:PEDOT-S solutions in different volume ratios like 3:1, 2:2, or 1:3 by magnetic stirring for 10 min. The resulting mixture was maintained at 4 °C for 2 h, which resulted in emulsions-gels. Further these samples are referred to as LPNF:GEL:PEDOT-S 24:8:1, LPNF:GEL:PEDOT-S 8:8:1, and LPNF:GEL:PEDOT-S 2.7:8:1. These are the relative ratios; however, all samples have the same total amount of protein. The resulting hydrogels were frozen in −80 °C overnight and lyophilized for 48 h to remove water to obtain the aerogels.

### 2.5 Rheology Properties of LPNF Matrix Hydrogels

Rheological tests were performed using a TA HR-2 rheometer equipped with 20 mm parallel stainless-steel plates. Rheological frequency sweeps from 0.1 to 100 rad s^–1^ were performed with a shear strain of 1%. Rheological measurements of dynamic strain sweep of the LPNF composite gels were performed at a constant frequency of 1 Hz over a strain range of 0.1–10000%. Samples were prepared in situ on the rheometer by mixing all components and quickly lowering the geometry. All samples were run in duplicates.

### 2.6 Specific Surface Area Analysis of LPNF Matrix Aerogels

The specific surface area of aerogels was measured at the relative pressure of *P*/*P*_0_ = 0.995 by the Brunauer–Emmett–Teller (BET) method on a specific surface and pore analyzer (Micromeritics ASAP 2020). The nitrogen isothermal adsorption and desorption volumes were measured at −196 °C with samples outgassed at 200 °C for 5 h. The pore size distribution was calculated from the adsorption isotherm using the Barrett–Joyner–Halenda method.

### 2.7 TGA Analysis of LPNF Matrix Aerogels

A thermogravimetric analysis (TGA) instrument (Mettler Toledo) was used to evaluate the thermal properties of the aerogel. The measurements were performed in the range of 30–900 °C with a heating rate of 10 °C/min in 900 μL alumina crucibles under nitrogen gas flow. Furthermore, to estimate the residue content of the aerogel, it was maintained at 900 °C with isothermal conditions for 30 min.

### 2.8 Young’s Modulus of LPNF Matrix Aerogels

The characterizations of Young’s modulus of LPNF matrix aerogels were performed by adding different weight on the aerogels and measuring the deformations. The Young’s modulus was then obtained as the slope from a plot of the stress against strain.

### 2.9 Conductivity Measurements

The sheet resistance of PEDOT-S was measured by the four-probe method (Jandel RM3000 station). Films were formed by spin-coating of PEDOT-S solutions. The film thickness was determined on a Dektak 6 M stylus profiler equipped with a 12.5 μm stylus tip from Veeco. Film thickness values were estimated by cutting the film with a scalpel and determining the maximum depth of the profile roughness below the mean line across such a cut.

The conductivity σ was calculated by using the formula
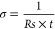
where *Rs* represents the sheet resistance of the films (*Rs* = 0.35 mΩ for PEDOT-S with Na^+^; *Rs* = 0.34 mΩ for PEDOT-S with K^+^), and *t* represents the thickness of the PEDOT-S films (*t* = 28 nm for PEDOT-S with Na^+^; *t* = 29 nm for PEDOT-S with K^+^).

The conductivities of aerogels were determined by electrical impedance spectroscopy. The aerogels were cut with a razorblade to ensure a smooth surface. The height of the cut aerogels plugs was 1 cm with a diameter of 1 cm. Two pieces of aluminum foil were glued with silver paste onto the aerogel surfaces. The following equation was used to calculate the conductivity σ:
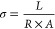
where *L* represents the height of aerogel, *R* represents the impendence value, and *A* represents the contact area between electrodes and the aerogel. Conductivity corrected by density was calculated according to the following equation:^60^
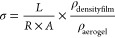


The density of solid film was 1.1 g/cm^3^, and the density of PEDOT-S in the aerogel was estimated by the mass of PEDOT-S added to each aerogel divided by aerogel volume.

### 2.10 Characterization of Piezoresistive Pressure Sensing

The aerogels were cut with a razorblade to ensure a smooth surface. The height of the cut aerogel plugs was 1 cm with a diameter of 1 cm. Two pieces of aluminum foil were glued with silver paste onto the aerogel surfaces and the electrodes were connected to a Keithley 2400 source meter. The compression tests were performed by adding (or removing) different weights onto the LPNF:GEL:PEDOT-S aerogel. The variations in current as a function of applied pressure were recorded in real time.

## 3 Results and Discussion

### 3.1 Fabrication of LPNF Matrix Hydrogel/Aerogel

**Figure 2:**
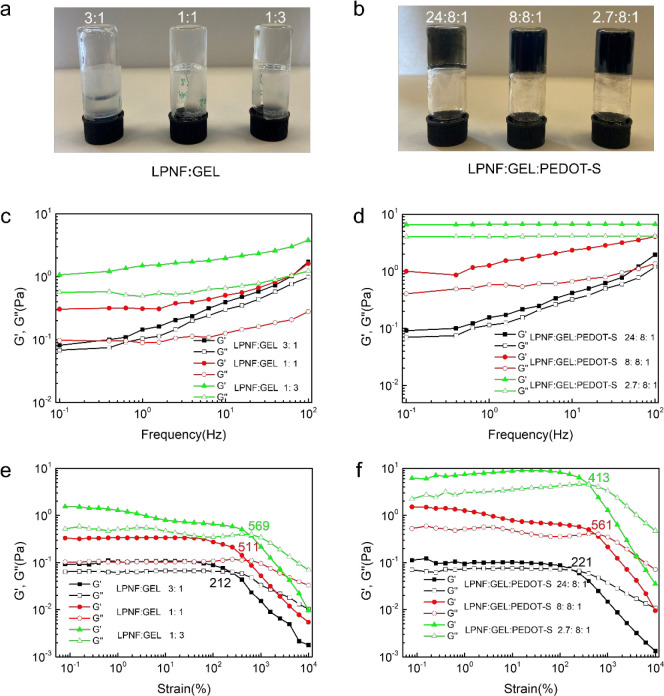
(a, b) Sol-to-gel transition of LPNF:GEL and LPNF:GEL:PEDOT-S gels by the test tube inverting method. (c) Rheological measurements of dynamic frequency sweep of the different ratios of LPNF with gelatin at a strain of 1% over a range of 0.1–100 Hz. (d) Rheological measurements of dynamic frequency sweep of the different ratios of LPNF with GEL:PEDOT-S at a strain of 1% over a range of 0.1–100 Hz. (e) Rheological measurements of dynamic strain sweep of the different ratios of LPNF with gelatin at a constant frequency of 1 Hz over a strain range of 0.1–10000%. (f) Rheological measurements of dynamic strain sweep of the different ratios of LPNF with GEL:PEDOT-S at a constant frequency of 1 Hz over a strain range of 0.1–10000%.

The method employed for fabrication of hydrogels and aerogels is outlined schematically in Figure 1. We first describe formation of hydrogels and their mechanical properties, and this is followed by a description of the process of conversion of the hydrogel into an aerogel and structural and mechanical properties of the aerogel with and without PEDOT-S. PEDOT-S was synthesized following a known procedure; however, two versions of PEDOT-S were prepared with Na^+^ or K^+^ as counterion (Supporting Information, Figure S1a). The two versions displayed similar properties and absorption spectra (Figure S1b,c). However, when attempting to form aerogels during preliminary investigations (see section 3.4 below for more discussion of the aerogels), it proved impossible to form homogeneous aerogels when employing the Na^+^ material. On the other hand, when employing the K^+^ material, aerogels with a homogeneous distribution were readily prepared. Photos of the two types of aerogels are provided in Figure S1d. In all of the discussions below, PEDOT-S refers to the version prepared with K^+^ as counterion. As the first step, aqueous solutions/dispersions of lysozyme protein nanofibrils (LPNFs) and gelatin (GEL) were prepared. LPNFs were prepared by heating a stirred acidic aqueous solution of hen egg-white lysozyme at 80 °C for 24 h. The GEL solution was prepared by dissolving gelatin at 60 °C in water. The LPNF dispersion and the GEL solution were then mixed in different volume ratios, which resulted in samples with LPNF:GEL ratios of 1:3, 1:1, and 3:1 (while ensuring that the total amount of protein remained constant, for example, the 1:1 sample was prepared by mixing LPNFs and GEL in a 2:2 ratio). The different samples were prepared by mixing warm PNF dispersions with warm GEL solutions, and the resulting mixtures were then allowed to cool to ambient temperature. The gelation properties of the mixtures were initially examined by the test tube inversion method. The different samples after cooling are shown in Figure 2a. Upon cooling, the GEL component will tend to gel, and accordingly a high GEL fraction will promote formation of strong gels. In the case of the LPNF:GEL 3:1 sample, an increase in viscosity is observed upon cooling; however, due to the relatively low concentration of gelatin, the resulting gel network is weak and the gel is not self-supporting. On the other hand, for samples with a higher fraction of GEL (LPNF:GEL 1:1 and 1:3), self-supporting gels are formed upon cooling, as apparent from the results shown in Figure 2a.

### 3.2 Effect of PEDOT-S on Gelation Properties

The effect of addition of PEDOT-S on the gelation properties of the LPNF:GEL system was investigated by preparing samples with ratios of LPNF:GEL:PEDOT-S of 24:8:1, 8:8:1, and 2.7:8:1 (i.e., corresponding to LPNF:GEL ratios of 3:1, 2.:2, and 1:3, respectively). The samples were then allowed to reach ambient temperature to induce gelation. When the gelation properties of the cooled samples were examined by the test tube inversion method, it was found that self-supporting gels formed in all cases. Accordingly, when PEDOT-S is introduced to the system, a strong hydrogel, that can support its own weight, is formed for all samples including the sample with a ratio of LPNF:GEL of 3:1 (that did not form a self-supporting gel in the absence of PEDOT-S, c.f. Figure 2a,b).

### 3.3 Rheological Properties

To obtain more quantitative information about the mechanical properties of the hydrogels, rheological measurements were performed for the various LPNF:GEL and LPNF:GEL:PEDOT-S gels. The samples were applied to the rheometer sample holder when warm and were then allowed to reach ambient temperature, to induce gelation, before the measurement. The storage (*G*′) and loss modulus (*G*′′) as a function of shear rate and strain were then measured for LPNF:GEL and LPNF:GEL:PEDOT-S samples as well as a GEL sample for comparison. First, dynamic oscillatory frequency sweep tests were carried out between 0.1 and 100 Hz at a strain of 1% (Figure 2c,d). At all investigated frequencies, the storage moduli are larger than the loss moduli (*G*′ > *G*′′), indicating the solid-like nature of the gels. However, the LPNG:GEL ratio strongly influenced the dependence of the moduli on frequency. In the case of the sample with the highest ratio of gelatin (LPNF:GEL, 1:3), *G*′ and *G*′′ exhibited a pronounced plateau in the frequency range of 0.1–20 Hz, which is similar to the behavior of the pure GEL hydrogel (Supporting Information, Figure S2). On the other hand, the samples with a higher ratio of LPNFs (3:1 and 1:1) only displayed a plateau below 1 Hz, indicating a poor mechanical stability of these gels. As might be expected from the previous vial tilting tests, the inclusion of PEDOT-S led to an increased gel strength (Figures 2d). Compared to the LPNF:GEL gels, the LPNF:GEL:PEDOT-S hydrogels exhibited increased moduli. In the case of the LPNF:GEL:PEDOT-S hydrogel with the highest ratio of gelatin, the strong gel character was indicated by frequency independence as well as a high *G*′ value.

In addition, strain sweep experiments were performed at a frequency of 1 Hz (Figure 2e,f). For a reference gelatin hydrogel, a breakage strain of 81% was observed (Supporting Information, Figure S2). The breakage strain of the LPNF:GEL hydrogels increased from 212% to 569% when the LPNF:GEL ratio was changed from 3:1 to 1:3. For the LPNF:GEL:PEDOT-S hydrogels, the breakage strain first increased from 221% (LPNF:GEL:PEDOT-S 24:8:1) to 561% (LPNF:GEL:PEDOT-S 8:8:1) and then decreased to 413% for the sample with the highest gelatin ratio (LPNF:GEL:PEDOT-S 2.7:8:1). This result indicated that the sample with the highest ratio of gelatin is more rigid than the other two samples. The rheological measurements indicate that addition of PEDOT-S leads to improved mechanical properties of the hydrogels. The difference in modulus values indicates the presence of different networks of micro/nanostructures inside the gels, potentially influencing their mechanical properties.^61^ The increase in modulus upon addition of PEDOT-S is potentially useful when attempting to forma aerogels employing a freeze-drying process. A stronger gel network might withstand deformation resulting from the growth of ice crystals in the hydrogel, whereas a weaker hydrogel may undergo extensive deformation as a result of ice crustal formation. This means that a stronger hydrogel may be beneficial for formation of a more elastic aerogel, as is indeed found when comparing aerogels formed by freeze-drying of LPNF:GEL and LPNF:GEL:PEDOT-S hydrogels. For a given GEL:PNF ratio, the hydrogel with PEDOT-S has a higher modulus, whereas after freeze-drying the aerogel incorporating PEDOT-S has a lower Young’s modulus (see section 3.4 and Table 1).

**Table 1:** Comparative Results of Biomass Original or Aged Aerogels

sample	density (g cm^–3^)	pore diameter (nm)	BET surface area (m^2^ g^–1^)	Young’s modulus (kPa)
GEL	0.028	377	6.7	not given
LPNF:GEL (1:3)	0.020	357	12.63	4.66
Aged LPNF:GEL (1:3)	0.019	332	8.4	4.86
LPNF:GEL:PEDOT-S (2.7:8:1)	0.019	359	14.37	2.94
Aged LPNF:GEL:PEDOT-S (2.7:8:1)	0.018	362	15.49	2.93

### 3.4 Fabrication of GEL:LPNF Aerogels

**Figure 3:**
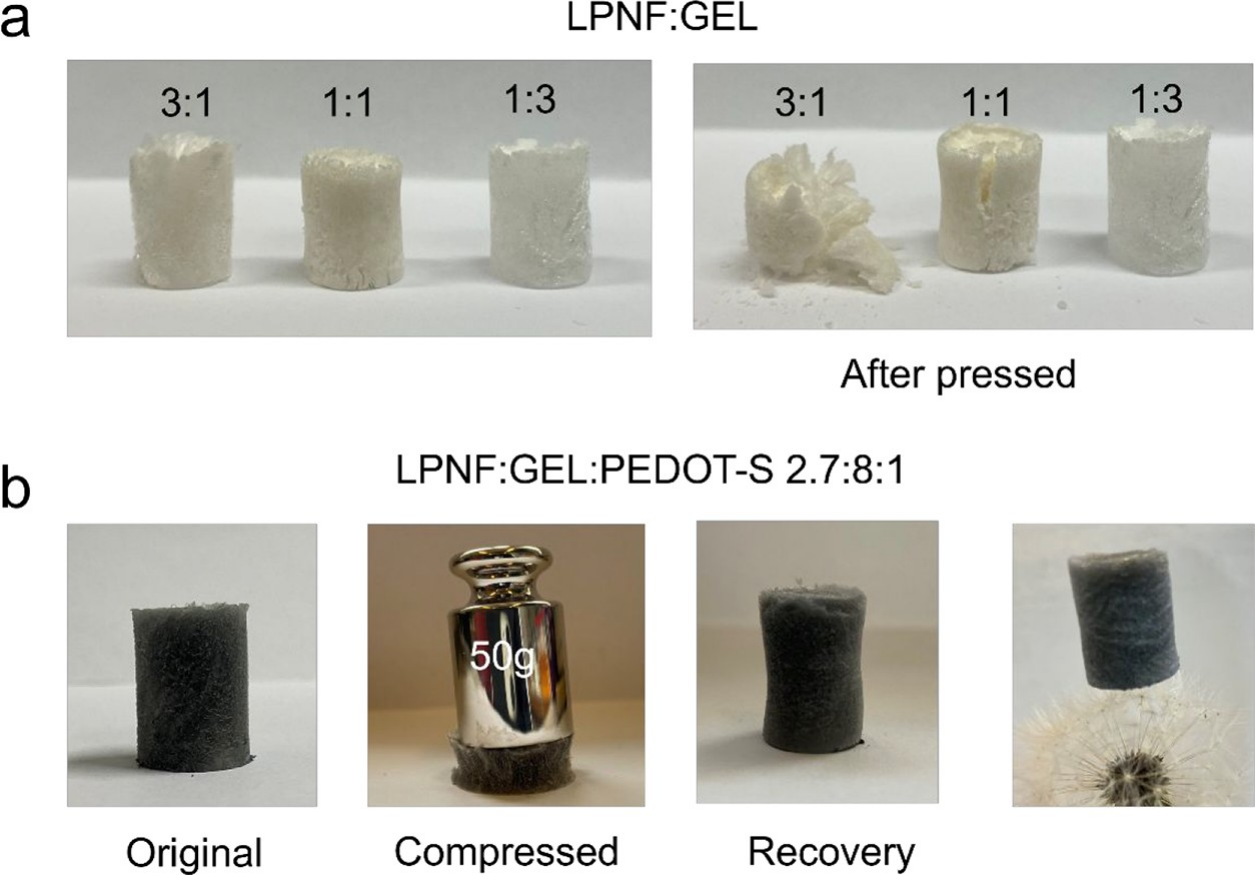
(a) Images of LPNF:GEL matrix aerogels before and after pressed. (b) Elastic lightweight LPNF:GEL:PEDOT-S composite aerogel.

As mentioned in the Introduction, a convenient way to prepare aerogels is through freeze-drying, where the hydrogel is first frozen followed by removal of water under reduced pressure. The different aerogels (LPNF:GEL 3:1; LPNF:GEL 1:1; LPNF:GEL 1:3) were frozen at −80 °C, followed by removal of the frozen water under reduced pressure. This resulted in the formation of aerogels for all samples. However, preliminary investigations showed that aerogels with a high ratio of LPNFs (i.e., the LPNF:GEL 1:1 and 3:1 samples) had less attractive mechanical properties than the LPNF:GEL 1:3 sample. As illustrated in Figure 3a, the LPNF:GEL 3:1 sample fractured when exposed to compression strain. The 1:1 sample showed elastic behavior, but upon compression cracks were formed, which resulted in permanent damage to the aerogel. Only the LPNF:GEL 1:3 sample underwent elastic deformation when exposed to compression forces. For these reasons, the studies described below focus on the LPNF:GEL 1:3 combination, with selected data also given for the other compositions. In addition, such gels functionalized with PEDOT-S displayed attractive elastic properties. For example, a LPNF:GEL:PEDOT-S aerogel can easily support a load ∼1700-times its own weight. In addition, the highly porous structure leads to ultralow apparent density and allows the lightweight composite aerogel to stand on a taraxacum officinalis as shown in Figure 3b.

#### 3.4.1 Structural Properties of LPNF:GEL Aerogels

**Figure 4:**
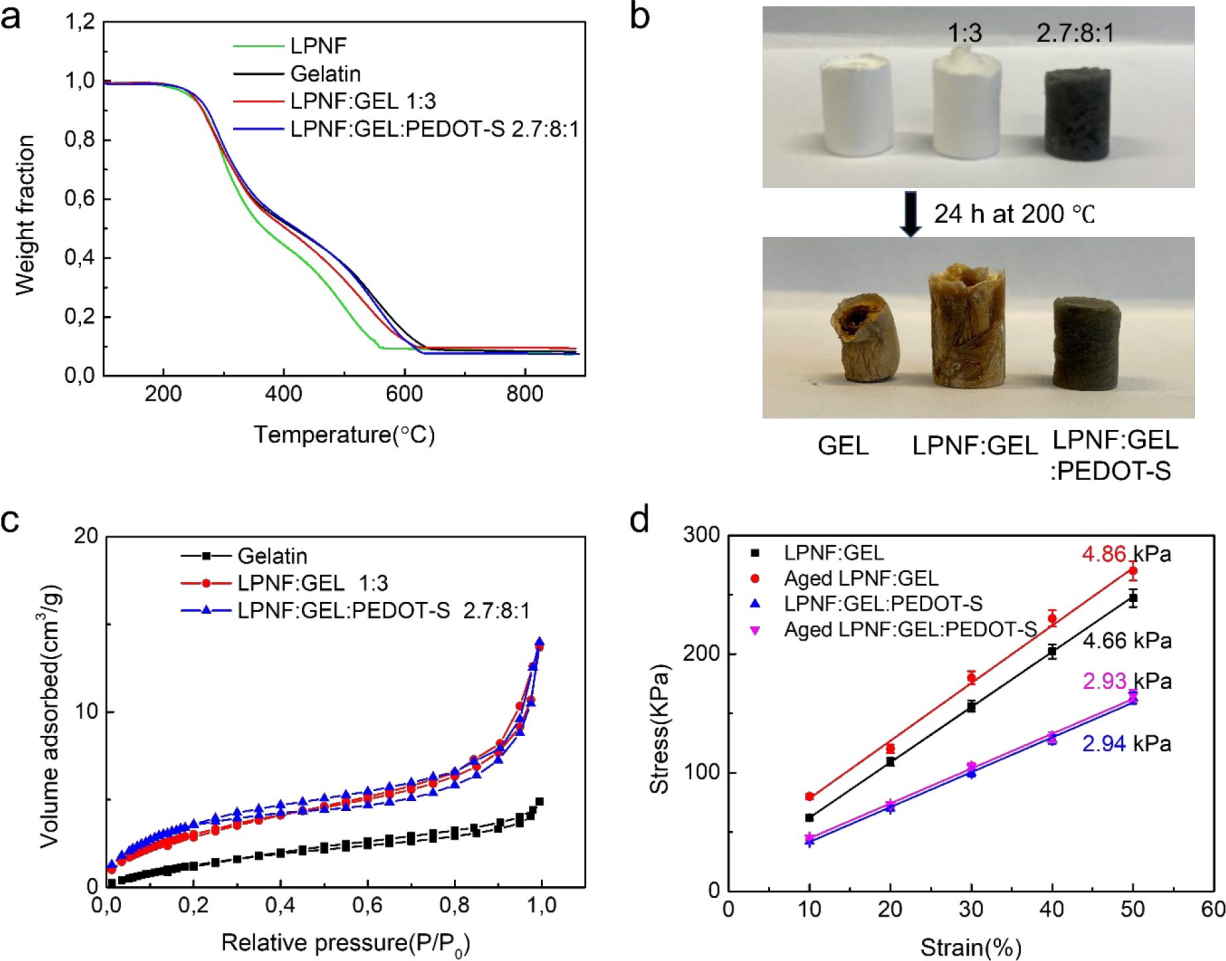
(a) TGA curves of amyloid fibril aerogel in nitrogen atmospheres. (b) GEL and LPNF matrix aerogels before and after aging for 24 h at 200 °C in air. (c) Nitrogen gas absorption–desorption curve of LPNF matrix aerogels. (d) Young’s modulus of LPNF matrix aerogels aged or not.

Structural characteristics and important parameters for the different aerogels are given in Figure 4 and Table 1. The thermal stabilities (under air) of LPNF:GEL and LPNF:GEL:PEDOT-S aerogels, as well as the GEL and LPNF components, were investigated by thermogravimetric analysis (TGA). As show in Figure 4a, all materials have similar thermograms; in all cases, material loss starts at about 270 °C. Upon reaching 600 °C, in all cases the samples have lost about 90% of their mass, the residual 10% most likely originating from inorganic residues such as salts, similarly to previously reported results.^62^ The thermogravimetric results indicate that the aerogels can withstand temperatures up to about 270 °C. This presents interesting opportunities for development of heat resistant aerogels where biopolymeric systems often can compete favorably with many polymeric materials derived from petrochemicals as reported in a recent study.^63^ We tested the thermal stability by heating the samples in air at 200 °C for 24 h. While the gelatin aerogel was deformed, the LPNF:GEL 1:3 gel underwent only slight deformation. However, in both cases, the samples were oxidized, which resulted in a brown color. The corresponding gel with PEDOT-S showed excellent thermal stability and was not deformed. In the discussion below, these types of samples that were heated at 200 °C for 24 h are referred to as aged samples.

The densities of the different aerogels (a reference sample made from 100% GEL, and samples made from LPNF:GEL 3:1; LPNF:GEL 1:1; LPNF:GEL 1:3 as well as the corresponding aerogels functionalized with PEDOT-S) are summarized in Figure S3 and Table 1. The reference gel prepared from pure gelatin has a density of 0.028 g cm^–3^. The density of the gels made from the different combinations of GEL, LPNFs, and PEDOT-S is similar for all the samples (0.020 g cm^–3^ for LPNF:GEL aerogels and 0.019 g cm^–3^ for LPNF:GEL:PEDOT-S aerogels). The density of aerogels incorporating LPNFs is thus significantly lower than the reference GEL aerogel. The LPNF:GEL and LPNF:GEL:PEDOT-S aerogels only underwent slight changes in density upon aging (at 200 °C for 24 h). The surface areas and pore volumes of the aerogels were investigated by N_2_ adsorption–desorption isotherm analysis by using the Brunauer–Emmett–Teller (BET) method (Figure 4c). The gelatin aerogel had a specific surface area of 6.7 m^2^ g^–1^, whereas when LPNFs were included (in a ratio of LPNF:GEL 1:3), the specific surface area increased to 12.63 m^2^ g^–1^. For the corresponding LPNF:GEL sample functionalized with PEDOT-S, the specific surface area increased even more to 14.37 m^2^ g^–1^. The average pore diameters of the GEL, LPNF:GEL, and LPNF:GEL:PEDOT-S aerogels were 377 nm, 357 nm, and 359 nm, respectively. Aging (200 °C for 24 h) of the LPNF:GEL led to a somewhat reduced pore size and a decrease in surface area. Aging of the LPNF:GEL:PEDOT-S aerogel did not significantly alter the pore size of and led to a slight increase in specific surface area (Table 1).

The LPNF:GEL aerogels have a Young’s modulus of 4.66 and 4.86 kPa before and after aging, respectively (Table 1 and Figure 4d). Functionalization with PEDOT-S resulted in a lower Young’s modulus: 2.94 and 2.93 kPa before and after aging, respectively (Table 1 and Figure 4d). The LPNF:GEL aerogel accordingly became stiffer after heat treatment, whereas the aerogel functionalized with PEDOT-S retained its softness after heat treatment. Moreover, the LPNF:GEL:PEDOT-S aerogel was softer than the corresponding LPNF:GEL aerogel, which is opposite to the trend observed during rheology measurements of the corresponding hydrogels (see section 3.3 and Figure 2c and d, comparing the green curves).

#### 3.4.2 Microstructure Morphologies of LPNF Matrix Aerogels

**Figure 5:**
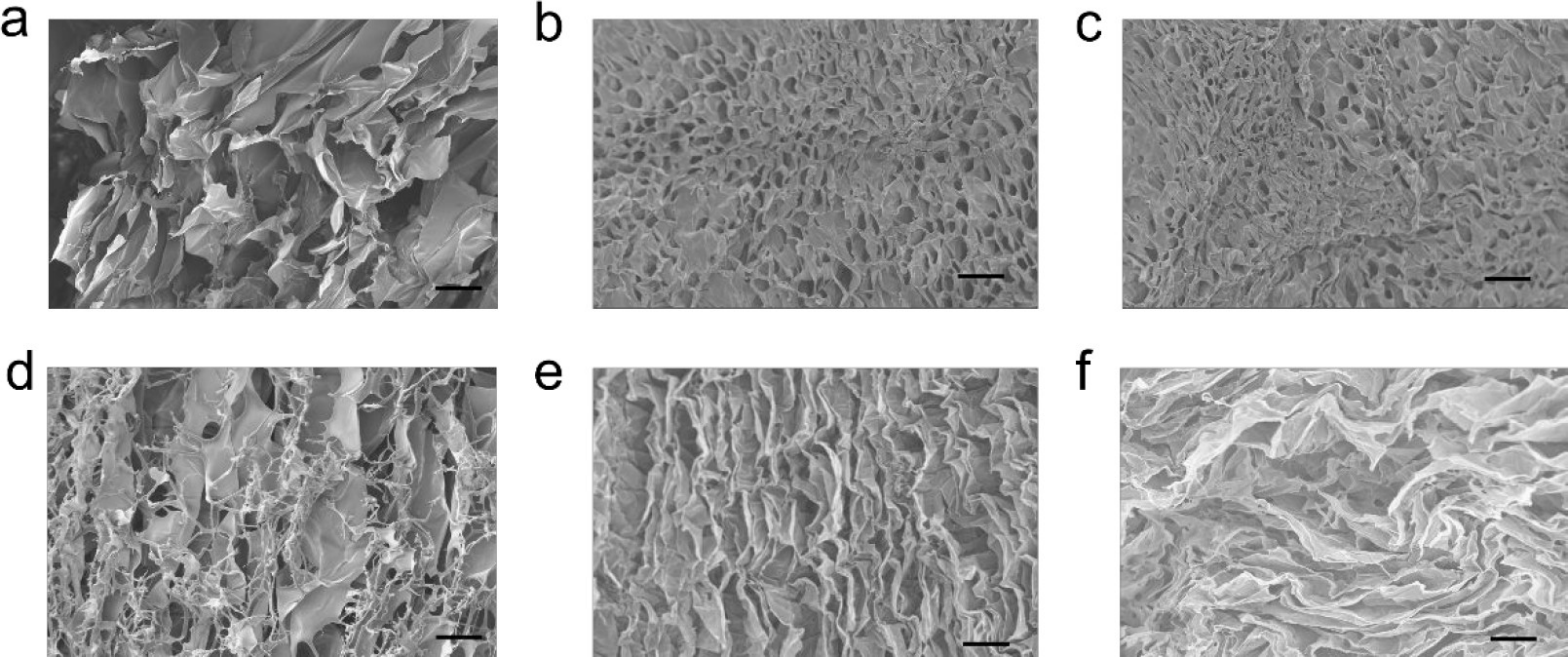
SEM images of aerogels. (a–c) Top view of LPNF:GEL (1:3), LPNF:GEL:PEDOT-S (2.7:8:1), pressed LPNF:GEL:PEDOT-S aerogel. (d–f) Cross-section of LPNF:GEL (1:3), LPNF:GEL:PEDOT-S (2.7:8:1), pressed LPNF:GEL:PEDOT-S. (f) Aerogels; scale bar is 10 μm.

The morphology of the aerogels was characterized by SEM, with typical images shown in Figure 5. The SEM images of LPNF based aerogels with a highly porous microstructure and interconnecting nanofibrous network can be clearly observed in all the aerogels. Figure 5a shows a typical structure obtained when imaging a LPNF:GEL aerogel from the top-side. The sample is made up of flake like structures, while when the cross-section of the sample is investigated, the sample has a flaky appearance with additional fiber shaped objects. The flake-like structure is a typical feature related to structural deformation due to growth of ice crystals when the sample is frozen. The incorporation of PEDOT-S has a dramatic effect on the sample morphology. When the sample is imaged from the top, honeycomb-like cells can be observed, while imaging of the cross-section displays sheet-like structures (Figure 5b,e). The formation of the honeycomb-like structures of the LPNF:GEL:PEDOT-S aerogel is likely to be related to the ability of the gel-network to withstand mechanical disruption during the growth of ice crystals during freezing of the sample.

The effect of deformation on the microstructure was investigated by imaging of a gel that had undergone repeated compression. The sample morphology is similar to that before deformation (Figure 5c,f). In the case of aged aerogels (heated at 200 °C), aerogels without PEDOT-S (LPNF:GEL) underwent structural changes, while in the case of aerogels with PEDOT-S (LPNF:GEL:PEDOT-S), the materials were somewhat compacted but with honeycomb-like structures preserved (Supporting Information, Figure S4).

As described earlier, the structural differences between aerogels prepared from GEL, LPNF:GEL, and LPNF:GEL:PEDOT-S can thus be rationalized by the hypothesis that additions of LPNFs to the GEL framework strengthens the gel and makes it less amenable to deformation from ice crystals that form when freezing the corresponding hydrogel. During freezing, the protein materials (and PEDOT-S) are accumulated among the side of ice crystals.^64^ The gel-network will be deformed during the growth of ice crystals and a stronger gel will able to counteract severe mechanical deformation. As observed during rheological measurements, addition of PEDOT-S leads to an increased gel strength, which is likely to be the origin of the large difference in microstructure observable between gels made with and without PEDOT-S. Gels with added PEDOT-S are able to withstand severe deformation of growing ice crystals and as a result form a network structure (Figure 5b,e). In contrast, for gels without PEDOT-S, it can be observed that the materials have been deformed giving flake-like structures.

## 4 Flexible Conductive Aerogels for Piezoresistive Pressure Sensing

**Figure 6:**
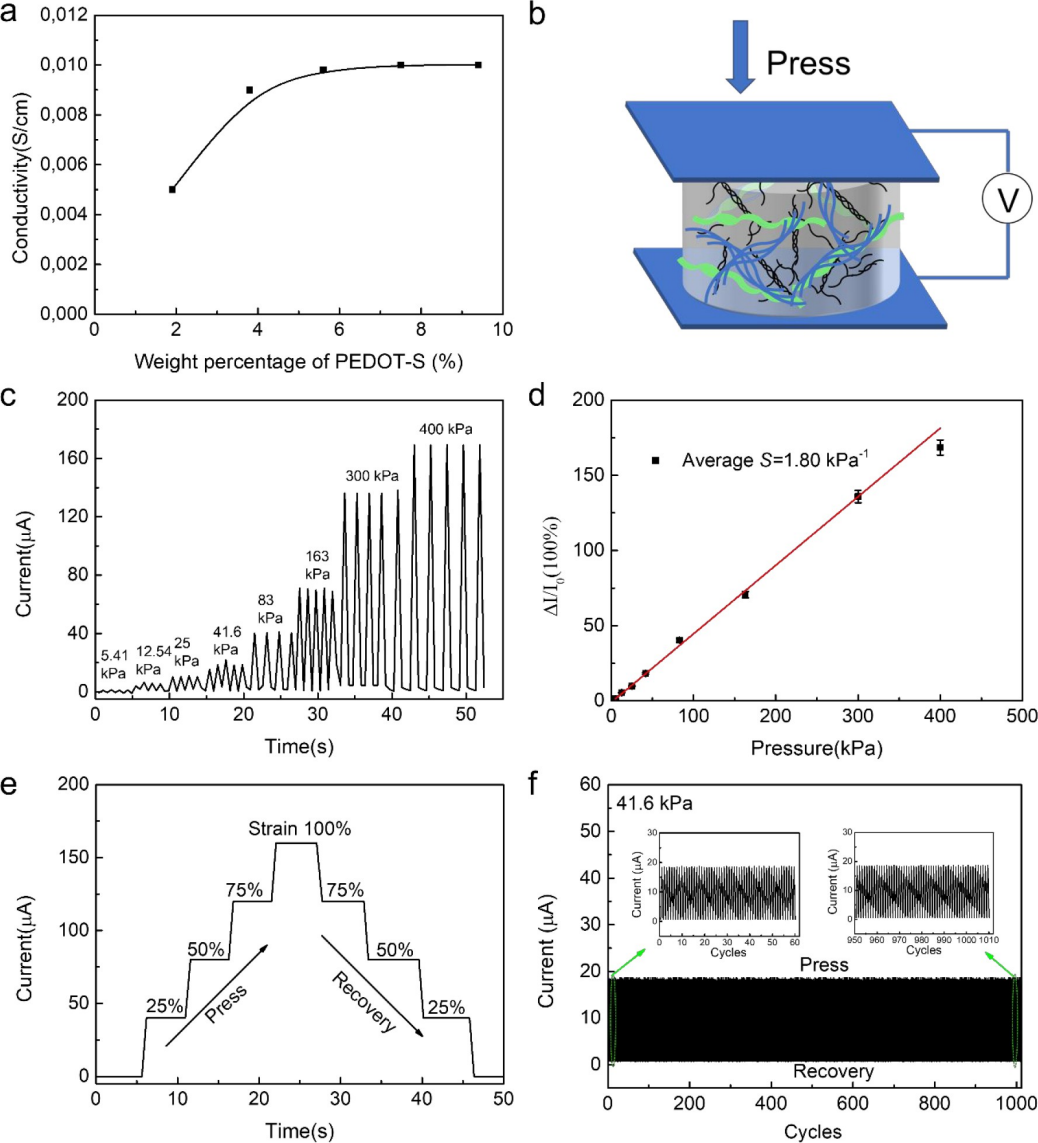
(a) Conductivity of LPNF:GEL:PEDOT-S matrix aerogels as a function of PEDOT-S concentration (wt %). (b) Schematic illustration of devices under different compress. (c) *I*–*t* curves of LPNF:GEL:PEDOT-S (2.7:8:1) aerogel under loading–unloading with different pressure. (d) Average sensitivity of five independently prepared LPNF:GEL:PEDOT-S (2.7:8:1) aerogels. For each aerogel, each measurement point is the average of three measurements. (e) *I*–*t* curves of LPNF:GEL:PEDOT-S (2.7:8:1) aerogel under different compressive strain. (f) Current–time (*I*–*t*) curves of LPNF:GEL:PEDOT-S (2.7:8:1) aerogel when repeatedly applying and removing a weight corresponding to a pressure of 41.6 kPa (1000 cycles).

PEDOT-S is an electronically conductive polyelectrolyte, and the aerogels functionalized with PEDOT-S may accordingly be electrically conductive. The electrical conductivity for a spin-coated PEDOT-S film was 1 S cm^–1^, which is similar to previous reports.^65^ The conductivities of aerogels were determined by electrical impedance spectroscopy. The aerogels were cut with a razorblade to ensure a smooth surface. The height of the cut aerogel plugs was 1 cm with a diameter of 1 cm. Two pieces of aluminum foil were glued with silver paste onto the aerogel surfaces. LPNG:GEL 1:3 aerogels were prepared, incorporating different amounts of PEDOT-S. It was found that the conductivity of the aerogels initially increased as the PEDOT-S concentration increased, eventually reaching a maximum value of 0.01 S cm^–1^ (Figure 6a), after which no increase in conductivity was observed. To estimate the intrinsic conductivity of PEDOT-S, this value can be corrected^60^ by comparing the estimated density of a pure PEDOT-S film (1.1 g cm^–3^) and the actual density of PEDOT-S in the aerogel (i.e added amount of PEDOT-S divided by the volume of the aerogel). If this correction is applied, the conductivity is about 2 S cm^–1^, which corresponds well the measurement of a reference PEDOT-S film (1 S cm^–1^) and with previous reports.^58^

To visually demonstrate the electrical conductivity of an aerogel, an LED was incorporated into the circuit. When a voltage of 3.5 V was applied to the uncompressed aerogel, the LED lit up, and as the pressure was increased, the light intensity increased (Supporting Information, Figure S5). In addition, the possibility of employing the conductive aerogel as a pressure sensor can be demonstrated through this type of setup. At 3.5 V, the luminance of the LED lamp increased as the LPNF:GEL:PEDOT-S aerogel was compressed due to the change in resistance upon compression.

To further characterize the piezoresistive performance of the conductive aerogel, quantitative data were obtained regarding the relationship between applied pressure and the observed current. A schematic outline of the experiment is shown in Figure 6b. We employed an LPNF:GEL:PEDOT-S (2.7:8:1) aerogel with a height of 1 cm with a diameter of 1 cm. The structural change (strain) induced by the pressure leads to a decrease of the resistivity of the aerogel thus influencing the current. Conversely, due to the elastic character of the aerogel, upon release of the applied pressure, the aerogel will return to the original state, leading to a return to the original resistance. A series of pressure–current relationship tests were carried out on the LPNF:GEL:PEDOT-S aerogel to explore its piezoresistive performance. The real-time current response (*I*–*t* curves) of LPNF:GEL:PEDOT-S aerogel as a function of pressure and strain was measured. As shown in Figure 6c and d, the current–pressure curve (*I*–*Pa*) of LPNF:GEL:PEDOT-S aerogel has an apparent linear relationship in a wide pressure range from 1.8 to 300 kPa with an average sensitivity of 1.80 kPa^–1^ (average of 5 independently prepared aerogels, see Table S1 and Figure S6), which is comparable to sensors developed from other biomass-derived conductive aerogels (summarized in Table 2). In Figure 6e is displayed the relation between strain and current. Under different compressive strain, the results in Figure 6e show that the LPNF:GEL:PEDOT-S aerogel can generate a stable current under an external constant force. The aerogels display a rapid change in current as a response to changes in the applied external force. The repeatability of pressure sensing was tested by applying and removing two weights (with the weights corresponding to a pressure of 41.6 and 163 kPa) for 1000 cycles and the device showed excellent repeatability at both pressures (see Figure 6f and Supporting Information Figure S7).

**Table 2:** Comparative Results of Biomass Conductive Aerogel Sensors

materials	sensitivity (kPa^–1^)	linear range (kPa)	conductivity (S/cm)	ref
PANI/BC/CH^a^	0.31	0.3–1.8	not given	(66)
PPy/C^a^	58.9	0–5	not given	(67)
PEDOT/PSS/CNF^a^	14.8	0–95% strain	0.0012	(68)
BC/MXene^a^	125.8	0.2–10	not given	(69)
aPANI/GA^a^	28.62	0–14	not given	(70)
PPy/PVA/BLG fibrils^a^	0.088	not given	0.042	(52)
LPNF:GEL:PEDOT-S	1.8	1.8–300	0.01	this work

a PANI/BC/CH: polyaniline/bacterial cellulose/chitosan. PPy/C: polypyrrole/cellulose. PEDOT/PSS/CNF: cellulose nanofibrils/poly(3,4-ethylene dioxythiophene)/poly(styrenesulfonate). BC/MXene: Bacterial Cellulose/Ti_3_C_2_T_*x*_ MXene. aPAN/GA: Alkali-treated Polyacrylonitrile/Graphene. PPy/PVA/BLG fibrils: Polypyrrole/poly(vinyl alcohol)/β-lactoglobulin fibrils. CB/PDMS: Carbon black/polydimethylsiloxane.

## 5 Conclusion

Herein we developed a protein-based elastic conductive aerogel with attractive mechanical properties. The employed proteins (gelatin and hen egg-white lysozyme) are of low cost, and conversion of lysozyme to lysozyme protein nanofibrils (LPNFs) involves simple self-assembly processes. The properties of the resulting hydrogels and aerogels are influenced by the ratio LPNFs to gelatin as well as the addition of the conductive polymer PEDOT-S. Upon freeze-drying, elastic electrically conductive aerogels are formed that can be employed as pressure sensors.

## Supporting Information Available

UV–vis data for PEDOT-S and photos of PEDOT-S functionalized aerogels; rheology data for gelatin samples; data of densities of different aerogels; SEM images of aged aerogels; resistivity test of aerogel with LED; additional characterization of conductive aerogels (PDF)
