# Supplementary material for: Protein-Based Flexible Conductive Aerogels for Piezoresistive Pressure Sensors
Source: ACS Appl Bio Mater. 2022 Jun 13;5(7):3360–70. doi: 10.1021/acsabm.2c00348 (PMC9297298; doi:10.1021/acsabm.2c00348)
Supplement: Supplementary file 1 — mt2c00348_si_001.pdf [file mt2c00348_si_001.pdf]

Supporting Information

Protein-Based Flexible Conductive Aerogels for Piezoresistive Pressure Sensors

Yusheng Yuan [a], Niclas Solin [a,*]

a: Department of Physics, Chemistry, and Biology, Biomolecular and Organic Electronics, Linköping University, 581 83 Linköping, Sweden

Email: niclas.solin@liu.se

1. PEDOT-S Synthesis and properties

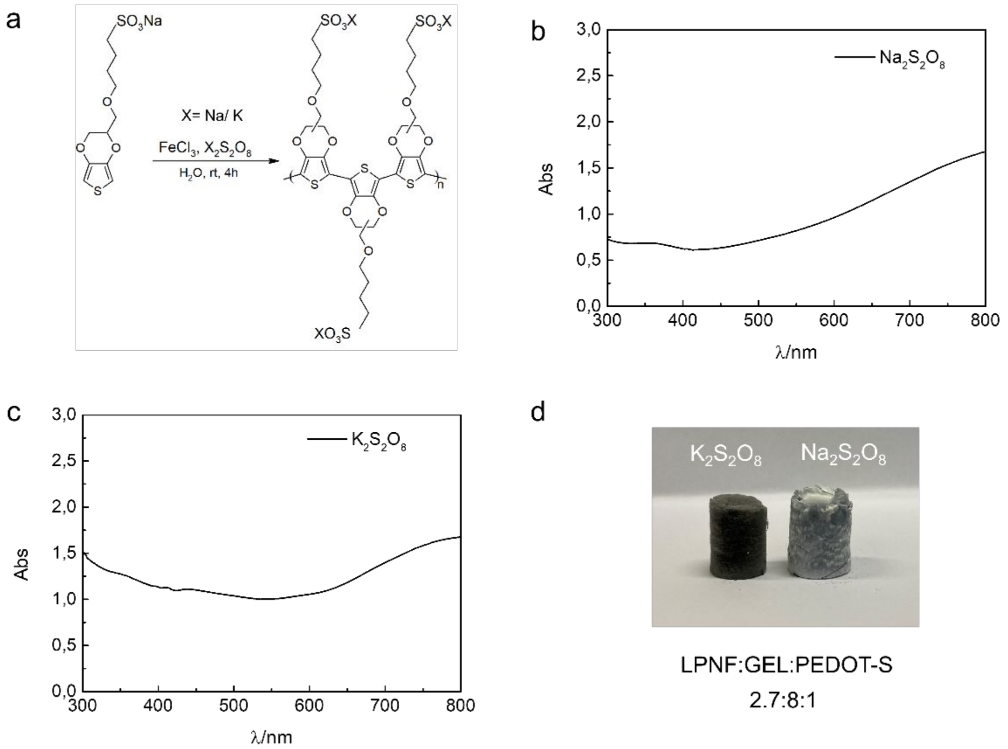

Figure S1 (a) Synthesis route of PEDOT-S.[1] (b) UV-vis absorbance spectra of dilute PEDOT-S (0.2 mg·mL^{-1}, synthesized by $Na_2S_2O_8$ agent) adjusted by Britton–Robinson buffer at pH 2 as indicated. (c) UV-vis absorbance spectra of dilute PEDOT-S (0.2 mg·mL^{-1}, synthesized by $K_2S_2O_8$ agent) adjusted by Britton–Robinson buffer at pH 2. (d) Different LPNF: GEL: PEDOT-S aerogel.

2. Rheology Properties of Pure Gelatin Hydrogels

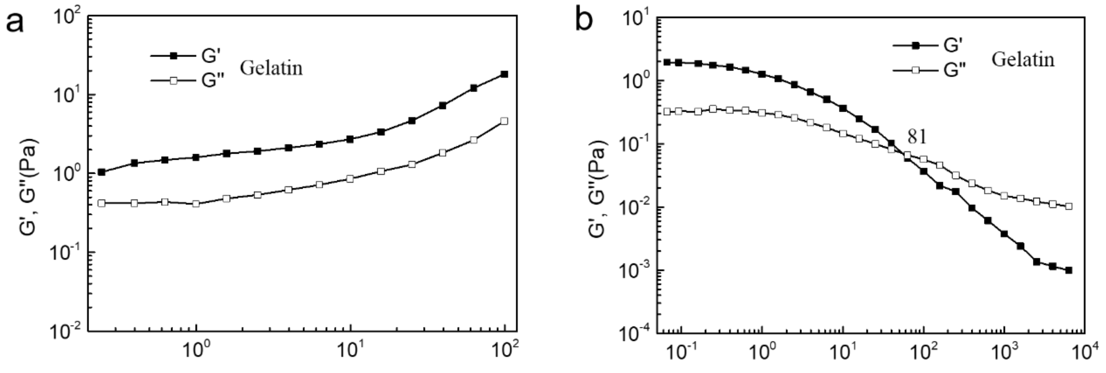

Figure S2 (a) Rheological measurements of dynamic frequency sweep of gelatin gel at a strain of 1% over a range of 0.1-100 Hz. (b) Rheological measurements of dynamic strain sweep of gelatin at a constant frequency of 1 Hz over a strain range of 0.1%–10000%.

3. Density of Different Aerogels

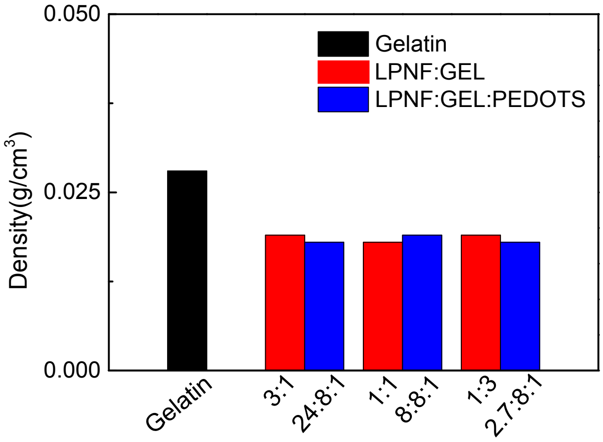

Figure S3 Density of the aerogels with the different ratio of LPNF and gelatin including PEDOT-S.

4. Microstructure Morphologies of Aged Aerogels

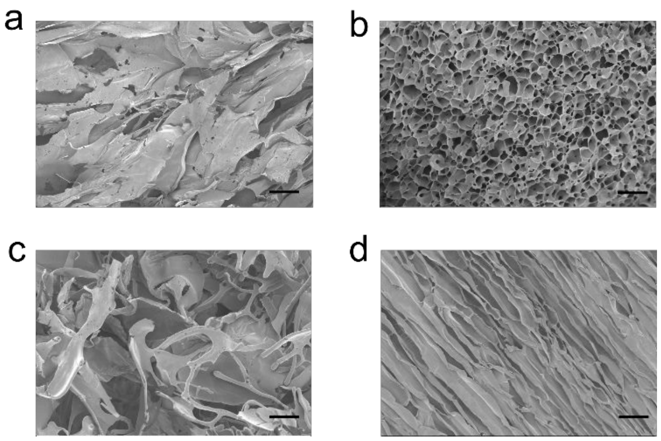

Figure S4 SEM images of aged aerogels. (a): Top view of LPNF: GEL (1: 3). (b): Top view of LPNF: GEL: PEDOT-S (2.7: 8: 1). (c): Cross-section of LPNF: GEL (1: 3). (d): Cross-section of LPNF: GEL: PEDOT-S (2.7: 8: 1). Scale bar is 10 μm.

5. Resistance of Flexible Conductive Aerogel

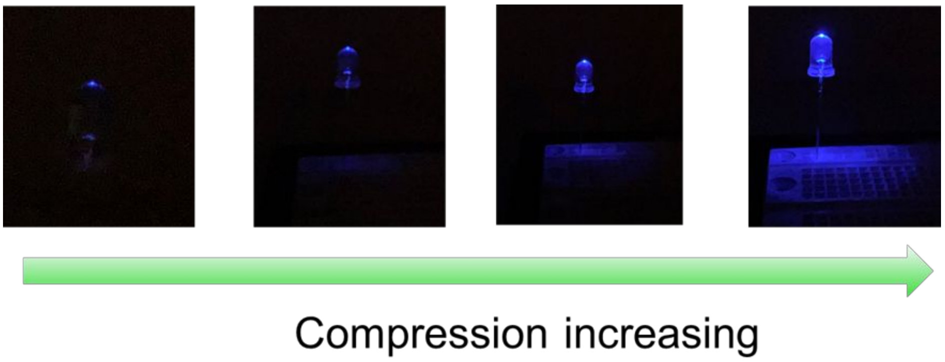

Figure S5 Luminance of the LED lamp increases as the LPNF: GEL: PEDOT-S aerogel is compressed

6. The Sensitivity of LPNF: GEL: PEDOT-S Aerogels

Table S1 The sensitivity of five independently prepared LPNF: GEL: PEDOT-S (2.7: 8: 1) aerogels within 1.8-300 kPa.

Sample	Sensitivity (kPa^{-1})
1	1.80 ± 0.09
2	1.82 ± 0.13
3	1.78 ± 0.23
4	1.81 ± 0.12
5	1.79 ± 0.14
Average	1.80 ± 0.21

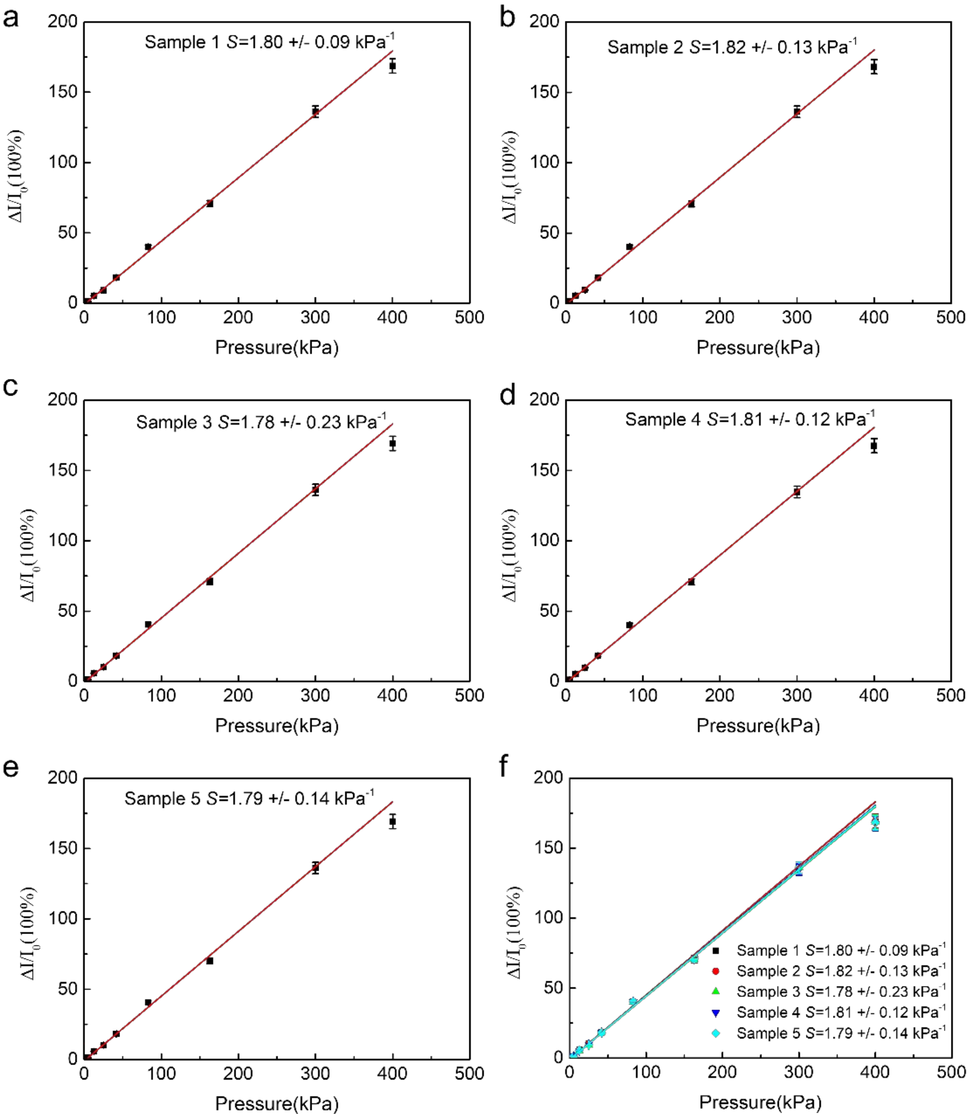

Figure S6 (a-e) The sensitivity of five independent LPNF: GEL: PEDOT-S (2.7: 8: 1) aerogels within 1.8-300 kPa, each sample was tested for triplicate. (f) The sensitivity of five independent LPNF: GEL: PEDOT-S (2.7: 8: 1) aerogels in one plot.

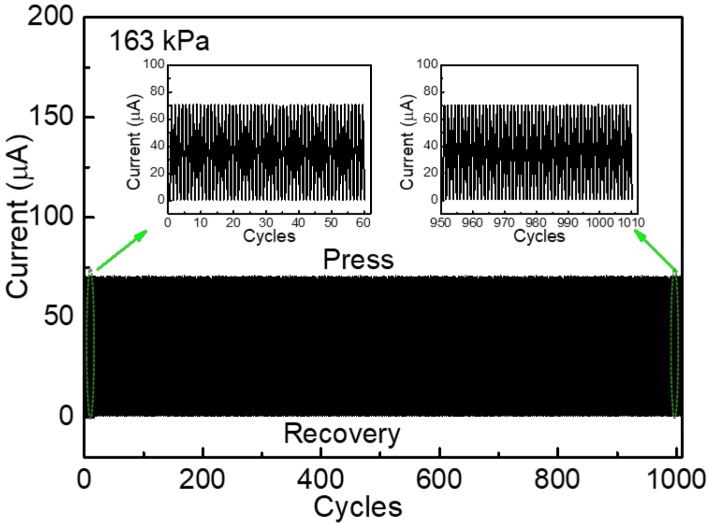

Figure S7 Current-time (I-t) curves of LPNF: GEL: PEDOT-S (2.7: 8: 1) aerogel after repeatedly applying and removing a weight corresponding to a pressure of 163 kPa (1,000 cycles).

References

(1) Karlsson, R. H.; Herland, A.; Hamedi, M.; Wigenius, J. A.; Åslund, A.; Liu, X.; Fahlman, M.; Inganas, O.; Konradsson, P. Iron-catalyzed polymerization of alkoxysulfonate-functionalized 3, 4-ethylenedioxythiophene gives water-soluble poly (3, 4-ethylenedioxythiophene) of high conductivity. *Chem. Mater*. **2009**, *21*, 1815-1821.
